# Genotype variation and genetic relationship among *Escherichia coli* from nursery pigs located in different pens in the same farm

**DOI:** 10.1186/s12866-016-0912-3

**Published:** 2017-01-05

**Authors:** Ana Herrero-Fresno, Shahana Ahmed, Monica Hegstad Hansen, Matthew Denwood, Camilla Zachariasen, John Elmerdahl Olsen

**Affiliations:** 1 Department of Veterinary Disease Biology, Faculty of Health and Medical Sciences, University of Copenhagen, Frederiksberg, Denmark; 2 Department of Large Animal Sciences, Faculty of Health and Medical Sciences, University of Copenhagen, Frederiksberg, Denmark

**Keywords:** *E. coli*, Swine, Genetic diversity, Genetic relationship, Antimicrobial selection, REP-PCR, PFGE

## Abstract

**Background:**

So far, little is known about the genetic diversity and relatedness among *Escherichia coli* (*E. coli*) populations in the gut of swine. Information on this is required to improve modeling studies on antimicrobial resistance aiming to fight its occurrence and development. This work evaluated the genotype variation of *E. coli* isolated from swine fecal samples at the single pig and pen level, as well as between pens using repetitive extragenic palindromic (REP) PCR fingerprinting and pulsed field gel electrophoresis (PFGE). The genetic diversity of strains collected from media supplemented with ampicillin or tetracycline was also investigated. Besides, the genetic relationship of strains within each pen, between pens, as well as among strains within each group isolated from media with or without antibiotic, was assessed.

**Results:**

REP-PCR patterns (*N* = 75) were generated for all the isolates (*N* = 720). Two profiles (REP_2 and REP_5) dominated, accounting for 23.7 and 23.3% of all isolates, respectively. At the pig and at the pen level, the number of different strains ranged from two to eight, and from 27 to 31, respectively, and multiple isolates from a single pen were found to be identical; however, in some of the pens, additional strains occurred at a lower frequency. *E. coli* isolates yielding different REP profiles were subjected to PFGE and led to 41 different genotypes which were also compared.

**Conclusions:**

Despite the presence of dominant strains, our results suggest a high genetic diversity of *E. coli* strains exist at the pen level and between pens. Selection with antibiotic seems to not affect the genetic diversity. The dominant REP profiles were the same found in a previous study in Denmark, which highlights that the same predominant strains are circulating in pigs of this country and might represent the archetypal *E.coli* commensal in pigs.

**Electronic supplementary material:**

The online version of this article (doi:10.1186/s12866-016-0912-3) contains supplementary material, which is available to authorized users.

## Background

It is well known that antimicrobial use in food animals leads to selection for antimicrobial resistant bacteria [1, 2]. This represents a global concern since the antimicrobial resistance can jeopardize future treatment of disease in the food animals [3]. Furthermore, it can lead to spread of resistant zoonotic bacteria to humans or transfer of resistance genes harbored in these bacteria to the human intestinal microbiota [4, 5] causing infections that may fail to respond to the standard antibiotic treatment, leading to prolonged illness, greater health care costs, and a higher risk of death [3].

Indicator bacteria such as *Escherichia coli* constitute a natural part of the intestinal microbiota of humans, pigs and other animals, and they are often used to study development of the resistance level caused by antibiotic treatment [6], including how treatment affects genetic diversity [7]. In recent years there have been great improvements in our ability to model how antibiotic treatment affects the intestinal microbiota [8–10]. As an example, a recent modeling study of the effect of tetracycline treatment on the evolution of the *E. coli* microbiota in pigs indicated that the genetic diversity would be diminished immediately after treatment [8]. The study highlights the fact that pig production under intense antibiotic treatment poses a greater threat to human health, not only because of the selection for resistant clones, but also due to the reduction in the sensitive ones, which cannot then compete with the resistant microbiota for transmission to humans. However, this and other studies suffer from lack of knowledge on how diversity varies naturally in the *E. coli* population in pigs, both between pen-mates and between pens in the same farm. Thus, there is a need to provide this information, in order to be able to improve our use of modeling studies in the fight against antimicrobial resistance.

The scope of this study was to investigate the natural genotype variation of *E. coli* in the gut of pigs sharing the same pen (single pig and pen level, respectively), and between pigs located in different pens, including how being resistant to tetracycline or ampicillin affected the diversity. The genetic relationship of *E. coli* strains within each pen, between pens, as well as, among isolates collected from media with or without antibiotic was assessed.

## Methods

### Collection and preparation of samples

The present study was conducted on 72 nursery pigs (3–4 weeks after weaning) located in four different pens (18 pigs per pen) from a Danish farm. The farm was selected from the catalogue of three Danish specialized veterinary pig practices from different regions of Denmark. Nursery pigs were not treated with any antimicrobial. Zinc oxide (2500 ppm) was administered into the feed for 14 days after weaning. This farm was included as one out of five farms in a previous study on genetic diversity of *E. coli* between farms [11]. Rectal fecal samples were collected from all the pigs by trained veterinarians in the spring of 2012 and were immediately cooled using ice packs and sent to the laboratory for analysis the following day. The fecal samples (ca. 1 g) were diluted in saline solution (0.9% NaCl) and plated on McConkey agar (Oxoid, Thermo Scientific, Roskilde, Denmark) without antibiotic, McConkey supplemented with ampicillin (25 μg/ml) and McConkey supplemented with tetracycline (25 μg/ml) incubated overnight at 37 °C to obtain *E. coli* strains. Antibiotics were purchased from Sigma (Sigma-Aldrich, Copenhagen, Denmark). CFU counts were performed from ten-fold serial dilutions.

### Bacterial isolation and identification

A total of 720 lactose positive, presumptive *E. coli* colonies were randomly selected from the 72 pigs. From each pig, four colonies were picked from the McConkey plate with no antibiotic, three from a plate with tetracycline and three from a plate with ampicillin (*N* = 10). The species was confirmed by the Microbact Gram negative identification system 24E™ (Oxoid) according to the manufacturer’s instructions.

### REP-PCR

Each colony was resuspended in 100 μl of sterilized milliQ water, boiled for 10 min and centrifuged for 5 min at 13000 rev/min. Quantity and quality of DNA was assessed by agarose gel-electrophoresis using standard techniques [12]. The primers used for the PCR reaction were Rep1R-I (5’-III ICG ICG ICA TCI GGC-3’) and Rep2-I (5’-ICG ICT TAT CIG GCC TAC-3’). DNA amplification was performed as previously described [11, 13] and the annealing temperature was 48 °C. The PCR reaction (25 μl) contained 50 ng of template DNA, 3.5 μl of each primer (10 μmol/l stocks), 3.5 μl of Dimethyl Sulfoxide (Sigma-Aldrich) and 13 μl of DreamTaq Green DNA Polymerase (Thermo Scientific, Roskilde, Denmark). A negative control (autoclaved milliQ water) was included in each PCR experiment. Genomic DNA of *E. coli* K-12 strain W3110 was used for the standardization of the REP-PCR reactions, to assess reproducibility and as a positive control [11]. GeneRuler 1-kb Plus molecular weight marker (Fermentas, Thermo Scientific, Roskilde, Denmark) was loaded into a well as an external reference standard. The PCR products underwent 1.2% gel electrophoresis for 3 h under constant 100 V, and visualized under UV-light after being stained with ethidium bromide (Carl Roth, Karlsruhe, Germany).

### Genomic macro-restriction pulsed-field gel electrophoresis (PFGE) analysis

Genomic DNA from isolates representing different REP profiles was independently typed by PFGE using XbaI restriction enzyme (New England Biolabs, BioNordika, Herlev, Denmark, 20000 U/ml; 2 h at 37 °C). The generated fragments were separated by PFGE, performed in the CHEF-DR III System (Bio-Rad Laboratories, Copenhagen, Denmark) under previously described conditions [14]. The strain *E. coli* 722-1505-26n EC was used as control (not published).

### Statistical analysis

The average number of different strains in each pig within different pens was compared using one-way ANOVA with multiple comparisons. A *p-value* <0.05 was considered statistically significant. BioNumerics version 7.5 (Applied Maths, Austin, USA) was used to analyze the REP and XbaI DNA fingerprints obtained for *E. coli* isolates. Digital REP-PCR and PFGE gel images were imported into BioNumerics and processed using the default settings. For REP profiles, each gel was normalized using 1-kb Plus DNA ladder, in the range of 75 to 20,000 bp as an external reference standard. For XbaI patterns, *E. coli* 722-1505-26n EC (not published) was used as reference. DNA fingerprint similarities were calculated using the curve-based Pearson coefficient with 1% optimization. Dendrograms were generated using the unweighted-pair-group method using arithmetic averages (UPGMA). For REP dendrograms, isolates of ≥92% similarity were treated as a single isolate. Clusters were considered at a 60% similarity cut-off and sub-clusters at 80% similarity [15, 16]. When studying the PFGE genotype diversity, a minimum similarity cut-off value of 70% was used to establish clusters [17].

The Shannon diversity index (*H′*) was used to calculate the genetic diversity of the *E. coli* isolates and was calculated as follows:

$$ H\hbox{'}=-{\displaystyle \sum_{i=1}^s}{p}_i \ln {p}_i $$

Here, *S* is the number of unique genotypes, and *p*_*i*_ is the number of isolates sharing the same genotype *i* over the total number of isolates [16]. This measure is also sometimes referred to as Shannon entropy, and is one of a spectrum of diversity measurements that differs in the extent to which the abundance of each genotype contributes to overall diversity [18].

We also investigated the diversity associated with data collected from a previous study from the same farm, where different REP profiles were assigned to 50 colonies and analyzed for each of the four pigs included in that work [11]. To examine the effect of sample size (number of colonies examined) on the diversity, the full dataset of 50 colonies from each of four pigs was non-parametrically bootstrapped (without replacement) to sample sizes between 1 and 50 with 10,000 bootstrap iterations. Both Shannon and Simpson’s diversity indices were calculated for comparison, with the latter calculated as follows:

$$ {\displaystyle \sum_{i=1}^s}{p}_i{{}^2}^{-1} $$

The bootstrapped distribution of diversity was obtained for each sample size and for each of the four individual pigs, as well as for the combined data from the four pigs.

## Results

### Quantification of *E. coli* in the different media

Results concerning the counts of total *E. coli,* as well as the counts of ampicillin and tetracycline resistant bacteria, in the fecal samples of every single pig were previously published [19] and are shown in Additional file 1. The average CFU/g of total *E. coli* (media without antibiotic) for pen 1, pen 2, pen 3 and pen 4 was 1.3 × 10^6^, 2.8 × 10^6^, 9.5 × 10^5^ and 2.3 × 10^6^, respectively. In media supplemented with antibiotic the obtained average CFU/g were: for tetracycline; 1.2 × 10^6^ (91.5% of total number of *E. coli* strains), 2.2 × 10^6^ (77.7%), 4.9 × 10^5^ (52,1%) and 1.4 × 10^6^ (58.3%), respectively, and for ampicillin; 3.1 × 10^5^ (23.5%), 3.6 × 10^5^ (12.9%), 4.8 × 10^5^ (50.1%) and 9.6 × 10^5^ (40.9%), respectively (Additional file 1).

### Genetic diversity of *E. coli* within a single animal and at the pen level

**Fig. 1:**
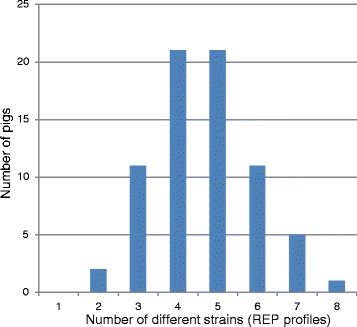
Genetic diversity of E. *coli* at the pig level. Number of different *E. coli* strains (REP profiles) versus number of pigs is shown

Ten strains from every single pig were analyzed to determine the extent of diversity within a single animal (without antibiotic selection and when selecting with ampicillin or tetracycline). The number of different patterns in a single pig varied from two (two pigs from pen 4) to eight (one pig from pen 1) (Fig. 1). The predominant numbers of different strains found in a single pig were four and five, both shown by 21 pigs, respectively (Additional file 2). REP_2 followed by REP_5 were the most common profiles, represented by at least one colony out of 10 colonies analyzed per animal, in most of the pigs (61 and 54 out 72, respectively) (Additional file 2). The average number of different strains per pig was 4.65 ± 0.27 and was not statistically different between the four pens analyzed (data not shown). Based on bootstrapped diversity measurements from previous data in the same farm (Additional file 3), the Shannon and Simpson’s diversity measures increased sharply up to approximately 10 colonies per pig, but then reached a plateau.

The genetic diversity of *E. coli* within each pen was analyzed to increase knowledge on strain variety that might be found between pen from a single farm under regular intensive pig farming conditions (Table 1). A total of 30, 31, 29 and 27 different REP profiles were observed in pens 1, 2, 3 and 4, respectively. REP_2 (44, 36, 42 and 49 strains from pen 1, 2, 3 and 4, respectively), REP_4 (46, 19, 36 and 12 strains), REP_5 (31, 57, 35 and 45 strains) and REP_17 (18, 24, 16 and 21 strains) were present in all of them. Some profiles were exclusively associated to a specific pen (Table 1).

**Table 1:** Genetic diversity of *Escherichia coli* from nursery pigs at the pen level

Pens	REP_profiles (N Colonies)
Pen 1	REP_2 (44), REP_ 3 (2), REP_4 (46), REP_5 (31), REP_6 (3), REP_9 (1), REP_17 (18), REP_ 18 (2), REP_19 (2), REP_28 (2), REP_30 (1)^a^, REP_31 (2)^a^, REP_32 (1)^a^, REP_35 (1)^a^, REP_38 (1), REP_39 (1), REP_45 (1), REP_53 (1), REP_56 (2)^a^, REP_57 (2)^a^, REP_58 (3)^a^, REP_59 (1)^a^, REP_60 (1)^a^, REP_61 (1), REP_62 (1)^a^, REP_63 (1), REP_70 (1), REP_72 (1), REP_74 (4)^a^, REP_75 (2)^a^
Pen 2	REP_2 (36), REP_4 (19), REP_5 (57), REP_ 6 (2), REP_10 (2), REP_17 (24),REP_18 (1), REP_19 (8), REP_24 (2), REP_27 (5)^a^, REP_28 (2), REP_29 (1), REP_34 (1)^a^, REP_39 (2), REP_42 (3), REP_47 (1)^a^, REP_48 (1)^a^, REP_49 (1)^a^, REP_ 50 (1)^a^, REP_51 (1)^a^, REP_ 52 (1)^a^, REP_53 (1), REP_54 (1)^a^, REP_55 (1)^a^, REP_63 (1), REP_69 (1)^a^, REP_70 (1), REP_71 (1)^a^, REP_ 72 (1), REP_73 (1)^a^
Pen 3	REP_2 (42), REP_3 (2), REP_4 (36), REP_5 (35), REP_10 (1), REP_17 (16), REP_18 (8), REP_19 (10), REP_20 (2), REP_21 (2), REP_22 (1)^a^, REP_23 (2)^a^, REP_24 (1), REP_25 (1)^a^, REP_26 (1)^a^, REP_33 (1)^a^, REP_36 (3), REP_38 (2), REP_40 (1)^a^, REP_41 (2)^a^, REP_42 (2), REP_43 (1)^a^, REP_44 (1)^a^, REP_45 (1), REP_46 (1)^a^, REP_63 (1), REP_66 (1)^a^, REP_67 (2)^a^, REP_68 (1)^a^
Pen 4	REP_1 (3), REP_2 (49), REP_3 (2), REP_4 (12), REP_5 (45), REP_6 (8), REP_ 7 (8)^a^, REP_8 (1)^a^, REP_9 (2), REP_10 (2), REP_11 (2)^a^, REP_12 (1)^a^, REP_ 13 (2)^a^, REP_14 (1)^a^, REP_15 (2)^a^, REP_ 16 (1)^a^, REP_17 (21), REP_18 (1), REP_19 (3), REP_20 (2), REP_21 (1), REP_36 (2), REP_37 (1)^a^, REP_38 (2), REP_61 (1), REP_64 (1)^a^, REP_65 (4)^a^

^a^profiles marked with ^a^were pen-specific

To depict the strain diversity and genetic relationship of *E. coli* isolates found within a pen, dendrograms of REP fingerprints were constructed by using the UPGMA method of tree building. Using the Pearson’s coefficient for comparison of REP fingerprints, similarity scores were generated; ranging from 20 to 98% for the isolates from pigs in pen 1, 20 to 96% for pen 2 isolates, 4.5 to 99% for pen 3 isolates and 5 to 82% for pen 4 isolates. The *E. coli* populations isolated from pen 1, pen 2, pen 3 and pen 4 samples were divided into 11, 12, 11 and 10 clusters, respectively (Table 2, Additional file 4). The *H′* diversity index calculated for *E. coli* obtained from each pen source was very similar (and differences were not statistically significant) and ranged from 2.28 (pen 1) to 2.37 (pen 3) (Table 2).

**Table 2:** Shannon diversity index of each group analyzed

Group/level	N isolates	N clusters	Shannon diversity index
Pen 1	180	11	2.28
Pen 2	180	12	2.27
Pen 3	180	11	2.37
Pen 4	180	10	2.34
Between pens	720	20	2.57
No antibiotic^a^	288	10	2.07
Ampicillin^a^	216	18	2.56
Tetracycline^a^	216	12	2.38

^a^No antibiotic, ampicillin and tetracycline denote whether colonies were obtained from plates without or with the mentioned antibiotics

### Strain diversity between pens

A total of 75 REP profiles were detected among the 72 pigs analyzed (Table 3). The dominant profile among the four pens was REP_2, represented by 171 strains (23.7% of the isolates analyzed), followed by REP_5 (168 isolates; 23.3%), REP_4 (113 isolates; 15.7%) and REP_17 (79 isolates; 11%). The rest of the profiles were represented by between 3.2% of the *E. coli* strains and 0.1% (corresponding to only one isolate from one specific pig). Approximately half of the profiles (46.1%) were represented by a single isolate (Table 3). In a previous study, including five Danish farms [11], REP_2 (there termed P4), REP_5 (there termed P3) and REP_17 (there termed P2) were also the most commonly found profiles.

**Table 3:** REP profiles detected in 72 nursery pigs from a Danish intensive pig farm

REP profile	N col (%)	REP profile	N col (%)	REP profile	N col (%)	REP profile	N col (%)
REP_1	3 (0.4%)	REP_21	3 (0.4%)	REP_41	2 (0.3%)	REP_61	2 (0.1%)
REP_2 (P4)^a^	171 (23.7%)	REP_22	1 (0.1%)	REP_42	5 (0.7%)	REP_62 (P24)^b^	1 (0.1%)
REP_3	6 (0.8%)	REP_23	2 (0.3%)	REP_43	1 (0.1%)	REP_63	3 (0.4%)
REP_4	113 (15.7%)	REP_24	3 (0.4%)	REP_44	1 (0.1%)	REP_64	1 (0.1%)
REP_5 (P3)^a^	168 (23.3%)	REP_25	1 (0.1%)	REP_45	2 (0.3%)	REP_65	4 (0.5%)
REP_6	13 (1.8%)	REP_26	1 (0.1%)	REP_46	1 (0.1%)	REP_66	1 (0.1%)
REP_7	8 (1.1%)	REP_27	5 (0.7%)	REP_47 (P32)^b^	1 (0.1%)	REP_67	2 (0.3%)
REP_8 (P24)^b^	1 (0.1%)	REP_28	4 (0.5%)	REP_48	1 (0.1%)	REP_68	1 (0.1%)
REP_9 (P26)^b^	3 (0.4%)	REP_29	1 (0.1%)	REP_49	1 (0,1%)	REP_69 (P31)^b^	1 (0.1%)
REP_10	5 (0.7%)	REP_30 (P25)^b^	1 (0.1%)	REP_50	1 (0.1%)	REP_70	2 (0.3%)
REP_11	2 (0.3%)	REP_31	2 (0.3%)	REP_51	1 (0.1%)	REP_71	1 (0.1%)
REP_12	1 (0.1%)	REP_32	1 (0.1%)	REP_52	1 (0.1%)	REP_72	2 (0.3%)
REP_13	2 (0.3%)	REP_33	1 (0.1%)	REP_53 (P27)^b^	2 (0.3%)	REP_73 (P29)^b^	1 (0.1%)
REP_14	1 (0.1%)	REP_34	1 (0.1%)	REP_54	1 (0.1%)	REP_74	4 (0.5%)
REP_15	2 (0.3%)	REP_35	1 (0.1%)	REP_55 (P28)^b^	1 (0.1%)	REP_75 (P35)^b^	2 (0.3%)
REP_16	1 (0.1%)	REP_36	5 (0.7%)	REP_56 (P33)^b^	2 (0.3%)		
REP_17 (P2)^a^	79 (11%)	REP_37 (P30)^b^	1 (0.1%)	REP_57	2 (0.3%)		
REP_18	12 (1.7%)	REP_38	5 (0.7%)	REP_58	3 (0.4%)		
REP_19	23 (3.2%)	REP_39	3 (0.4%)	REP_59	1 (0.1%)		
REP_20	4 (0.5%)	REP_40	1 (0.1%)	REP_60	1 (0.1%)		

*N col* number of colonies showing this profile

^a^profiles marked with ^a^were also found to be the dominant ones in a previous study on genetic diversity of *E. coli* between different pig farms [11]

^b^profiles marked with ^b^were exclusively detected in this farm in our previous study [11]

**Fig. 2:**
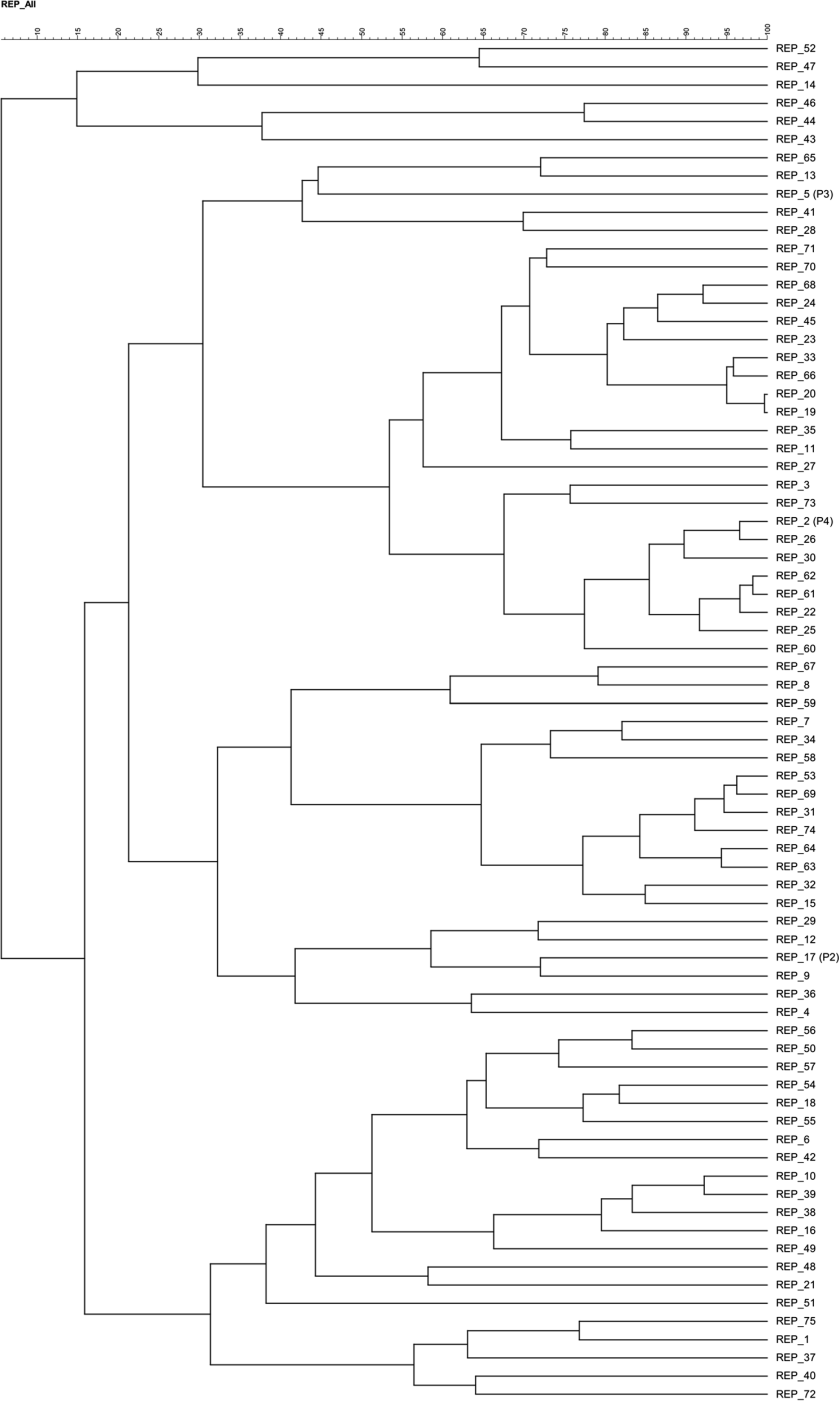
Dendrogram showing the relatedness of *E. coli* strains collected from the 72 pigs isolated in four pens as determined by REP-PCR. A condensed dendrogram (using a 60% cut-off value) is shown and contains 20 major clusters. DNA fingerprint similarities were calculated by the curve-based Pearson coefficient, and dendrograms were generated by UPGMA. Dominant profiles previously detected in this farm [11] are indicated between brackets

A composite dendrogram including all the unique fingerprints (*N* = 75) from all pens was also obtained. When the dendrogram was collapsed at 60% similarity cut-off value [15], it generated 20 clusters (Fig. 2). Among different clusters, cluster eight contained the largest number of profiles (*N* = 12) with DNA fingerprint similarities ranging from 68 to 99%, followed by cluster nine (22 profiles, 62 to 91% similarity), cluster 12 (11 profiles, 65 to 96% similarity) and cluster 10 (10 profiles, 68 to 98% similarity). The remaining 17 clusters comprised fewer than 10 profiles with fingerprint similarities ranging from 60 to 91%. The dominant profiles; REP_2, REP_5 and REP_17 were included in different clusters (cluster 9, cluster 6 and cluster 12, respectively). In general, the DNA fingerprint similarities observed ranged from 5 to 99% with an overall Shannon diversity index of 2.57 (Table 2).

### Analysis of the effect of antimicrobial resistance on strain diversity

The diversity in each group of strains able to grow in the presence of antibiotic (ampicillin or tetracycline) was also analyzed (Table 4). Of the total number of REP profiles detected, 36.8% represented isolates obtained from plates without antibiotics, 53.9% isolates obtained from plates with ampicillin, and 42.1% isolates from plates with tetracycline, despite more colonies (four per pig) were collected and analyzed from media without antibiotic versus three colonies from each of the plates with antibiotics. With this in mind, although statistically non-significant, the highest diversity was observed among the strains collected from media supplemented with ampicillin (41 out of the 75 REP profiles detected) and an *H*′ index of 2.57, followed by the tetracycline group (*H*′ = 2.38) and the group where no antibiotic was added to the media (*H*′ =2.01) (Table 2). The same, already mentioned profiles, REP_2, REP_4, REP_5 and REP_17 were the dominant ones in each of the groups, suggesting that they represent strains that are both ampicillin and tetracycline resistant (Table 4). Although at a very low frequency, a total of 15, 23 and 18 different REP profiles were exclusively found when no selection was performed and when selecting with either ampicillin or tetracycline, respectively. Six profiles were shared only among the ampicillin and tetracycline strains (Table 4). Individual dendrograms for strains isolated from media without antibiotic or containing ampicillin or tetracycline were also constructed. A total of 10, 18 and 12 clusters were observed for each of the groups (Table 2, Additional file 5).

**Table 4:** Genetic diversity depending on antimicrobial selection

Selection/N col	REP profiles (N col/N pigs)
No antibiotic/288	REP_2 (41/21), REP_3 (4/2), REP_4 (64/25), REP_5 (92/45), REP_9 (2/2), REP_17 (43/25), REP_18 (3/3), REP_19 (3/2), REP_28 (1/1), REP_33 (1/1)^a^, REP_34 (1/1)^a^, REP_36 (3/1), REP_38 (2/2), REP_39 (3/3)^a^, REP_61 (1/1), REP_63 (2/2), REP_64 (1/1)^a^, REP_65 (4/3)^a^, REP_66 (1/1)^a^, REP_67 (2/1)^a^, REP_68 (1/1)^a^, REP_69 (1/1)^a^, REP_70 (2/2)^a^, REP_71 (1/1)^a^, REP_72 (2/2)^a^, REP_73 (1/1)^a^, REP_74 (4/1)^a^, REP_75 (2/1)^a^
Ampicillin/216	REP_2 (66/39), REP_3 (2/1), REP_4 (7/5), REP_5 (46/25), REP_6 (10/8), REP_10 (4/4), REP_15 (1/1), REP_17 (22/15), REP_18 (6/4), REP_19 (5/5), REP_20 (2/1), REP_21 (2/2), REP_24 (1/1), REP_28 (1/1), REP_36 (2/1), REP_37 (1/1)^a^, REP_38 (3/3), REP_40 (1/1)^a^, REP_41 (2/2)^a^, REP_42 (5/3)^a^, REP_43 (1/1)^a^, REP_44 (1/1)^a^, REP_45 (2/2)^a^, REP_46 (1/1)^a^, REP_47(1/1)^a^, REP_48 (1/1)^a^, REP_49 (1/1)^a^, REP_50 (1/1)^a^, REP_ 51 (1/1)^a^, REP_52 (1/1)^a^, REP_53 (2/2)^a^, REP_54 (1/1)^a^, REP_55 (1/1)^a^, REP_56 (2/1)^a^, REP_57 (2/1)^a^, REP_58 (3/2)^a^, REP_59 (1/1)^a^, REP_60 (1/1)^a^, REP_61 (1/1), REP_62 (1/1)^a^, REP_63 (1/1)
Tetracycline/216	REP_1 (3/2), REP_2 (64/36), REP_4 (42/17), REP_5 (30/22), REP_6 (3/3), REP_7 (8/4)^a^, REP_8 (1/1)^a^, REP_9 (1/1), REP_10 (1/1), REP_11 (2/1)^a^, REP_12 (1/1)^a^, REP_13 (2/1)^a^, REP_14 (1/1)^a^, REP_15 (1/1), REP_16 (1/1)^a^, REP_17 (14/12), REP_18 (3/2), REP_19 (15/11), REP_20 (2/2), REP_21 (1/1), REP_22 (1/1)^a^, REP_23 (2/1)^a^, REP_24 (2/2), REP_25 (1/1)^a^, REP_26 (1/1)^a^, REP_27 (5/2)^a^, REP_28 (2/2), REP_29 (1/1)^a^, REP_30 (1/1)^a^, REP_31 (2/1)^a^, REP_32 (1/1)^a^, REP_35 (1/1)^a^

*N col* number of colonies, *N pigs* number of pigs

^a^profiles marked with ^a^were specific for each of the groups of isolates (collected from media without antibiotic or supplemented with ampicillin or tetracycline)

### Analysis of the genetic relatedness of the different REP profiles by PFGE

**Fig. 3:**
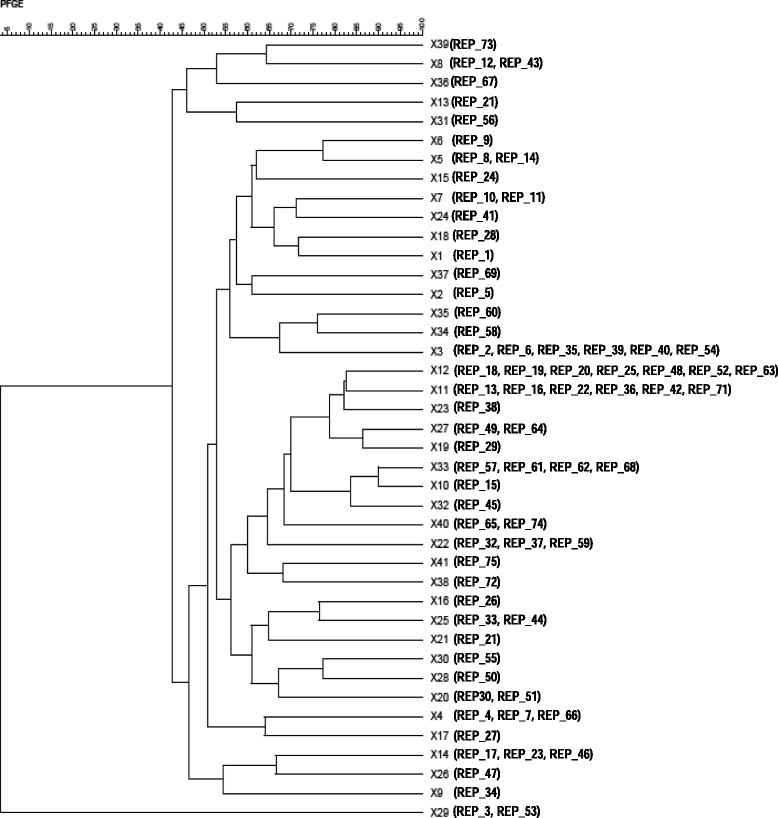
Dendrogram showing the relatedness between the 41 XbaI profiles derived from the 75 different *E. coli* strains (REP profiles) found in this study. At a cut-off value of 70%, 22 major clusters were obtained. REP profiles associated to each of the XbaI patterns are indicated between brackets

*E. coli* isolates assigned to the different REP profiles (one per profile) were further subjected to macro-restriction analysis with XbaI followed by PFGE. Using this method, they could be distributed into 41 XbaI profiles with X12 (comprised of seven REP profiles), X11 and X3 (comprised of six different REP profiles each) and X3 and X33 (comprised of four REP profiles each) as the most commonly observed (Fig. 3). The XbaI profiles were used to construct a dendrogram of similarity (Fig. 3) in order to investigate the genetic relatedness. At a cut-off value of 70% [17], the profiles were divided into 22 clusters (Fig. 3). Cluster 14 was the most common (nine isolates representing the profiles; X12, X11, X23, X27, X19, X33, X10, X32 and X40) and was the one encompassing the highest number of REP profiles (*N* = 25; 33.3%) (Fig. 3).

## Discussion

*E. coli* represents the predominant aerobic organism in the gut of pigs and other vertebrates, living in symbiosis with its host [20]. So far, little is known about the genetic diversity of this naturally occurring species in the intestine of swine. The genetic structure of commensal *E. coli* is determined by multiple host and environmental factors [20]. Studies on genetic diversity may allow a better characterization of the commensal niche and contribute to a better understanding of the genetics of these populations and their spread, as well as improve the design of modeling studies.

The main goal of the present study was to investigate how the genetic diversity of *E. coli* in fecal samples differs between pigs sharing the same environment, i.e., located in the same pen or in different pens in the same Danish farm. As well, we analyzed whether bacteria that were selected to be resistant to ampicillin or tetracycline would represent a more narrow (less genetically diverse) population.

REP-PCR was used to assess diversity. This genomic fingerprinting technique was chosen for several reasons; i) it generates specific strain patterns obtained by the amplification of repetitive DNA elements present along the *E. coli* genome [21], ii) the technique has proved more discriminatory than 16S rRNA PCR methods and restriction fragment length polymorphism [22, 23], and provides discriminatory power similar to randomly amplified polymorphic DNA (RAPD) [24]. In addition, the REP protocol is simpler and allows handling of a larger number of samples than other genomic DNA protocols, such as pulsed field gel electrophoresis, for molecular typing [22]. In addition it allowed us to compare our results to a previous study on genetic diversity of *E. coli,* where this technique was used [11].

We analyzed 10 colonies per animal, which according to the statistical approach performed (Additional file 3) might be representative of the *E. coli* diversity of each pig. Results from this study indicate that a single pig generally harbors one predominant strain of *E. coli* accompanied by one to a few other strains as previously observed in single animals such as human, gull and as previously demonstrated in pigs [11, 25]. While this limited number of different strains was observed at the animal level, REP-PCR DNA fingerprint analysis revealed extensive genetic diversity among *E. coli* strains isolated from different pigs regardless of whether they shared the same pen or not. The high genetic diversity displayed by *E. coli* was also demonstrated in humans and other animals such as cows, coyotes, sheep and goats in previous studies [16, 26–28].

The target farm of this work was also included in a previous study, encompassing five Danish farms, where fecal samples from four nursery pigs per farm were collected and 50 *E.* coli colonies per pig were analyzed by REP-PCR in order to assess the genetic diversity [11]. Among the five farms, this particular one (termed farm 4 in the previous study) was unique because nursery pigs did not receive antimicrobial treatment (they were treated with Zinc oxide only) and, therefore, it was selected to perform the present work. For this specific farm, 21 different REP profiles were observed, 12 exclusively found in this farm, being the one (out of the five analyzed) showing the highest diversity. In the current study, a total of 75 different REP profiles were observed, showing that the number of different strains increases when the total number of pigs and colonies is higher (72 pigs and 720 colonies). In our previous study, we speculated that the higher diversity observed in this farm compared to the rest of the farms was due to the lack of antimicrobial treatment of the pigs over the nursery period (only Zinc oxide was administered). However, in contrast to this premise, in the current work, when analyzing the effect of antimicrobial pressure selection on genetic diversity, it was observed, that apparently the majority of profiles are represented by strains that are resistant to either ampicillin or tetracycline or both. It is indicated to study how treatment affects diversity of the microbiota, but apparently even pigs that have not undergone treatment carry a high proportion of resistant strains, probably because such strains are wide spread in the pig industry.

To our knowledge, there is no information on how Zinc oxide might affect the *E. coli* diversity in the gut of pigs, and, since all the pigs in this and our previous study were treated with Zinc oxide, we cannot determine whether administration of this product could have an impact on diversity.

Even though a high diversity was observed, among the 75 different REP profiles obtained, four profiles were found to be very dominant and to represent 73.7% of the total number of strains analyzed. Therefore, we interpret these strains to be the archetypal commensal *E. coli,* and it would be of great relevance to sequence the isolates in order to identify common factors that set them aside from the other less frequent types. This is supported by the fact that the same dominant profiles REP_2 (23.7%), REP_5 (23.3%) and REP_17 (11%), designated; P4 (24.5%), P3 (28%) and P2 (16%), respectively, were observed in our previous study and at similar proportions. It has been reported that a strong selection takes place following excretion into the environment and that certain *E. coli* types can form stable populations [29] which could explain the establishment of dominant *E. coli* genotypes. Other factors such as a common diet may also explain the occurrence of dominant clones [29, 30].

The different REP profiles were represented by a total of 41 XbaI patterns, and also here a few patterns dominated. These results demonstrated that there is not a correlation between REP profiles and PFGE patterns and emphasize the importance of considering the resolving power of the technique being used when assessing strain diversity as previously demonstrated [17].

## Conclusions

In conclusion; this study was carried out to study the genetic diversity and relatedness among *E. coli* strains in the gut of nursery pigs*.* Overall we found REP_ 2, REP_5 and REP_17 to be the most common profiles among all the isolates, in accordance with a previous study [11]. These findings strongly suggest that these strains are the ones predominantly circulating in Danish farms and might be the archetypal commensal *E. coli* in the gut of pigs. Even though some isolates were predominant, and a limited number of profiles were found at the single animal level, we observed a high genetic diversity among *E. coli* strains isolated from different pigs regardless of whether they shared the same pen or not as previously demonstrated in other hosts [16, 26–28]. We also showed that apparently the majority of profiles were represented by strains that are resistant to either ampicillin or tetracycline or both. A correlation between REP and PFGE profiles was not detected highlighting the relevance of choosing the appropriate technique when analyzing diversity. Here, we suggest a good approach to assess diversity: REP-PCR for strain typing followed by PFGE analysis to study the relationship between the REP-PCR patterns.

### Availability of supporting data

We have not deposited additional or supporting data online. We show data relevant for the present study in the Additional files 1, 2, 3, 4 and 5.

## Additional files

Additional file 1: CFU/g of *E. coli* in the fecal samples of pigs. (XLSX 18 kb)
Additional file 2: Genetic diversity found in each single pig analyzed in this study. 10 colonies were isolated from each animal (four from media without antibiotic and three from media with ampicillin or tetracycline, respectively). 18 pigs per pen (*N* = 4) were included in the study. Number of colonies assigned to every REP profile detected is indicated between brackets. (TIFF 662 kb)
Additional file 3: The distribution of *E. coli* diversity obtained using a non-parametric bootstrap procedure to calculate diversity of a randomly chosen subset of 50 colonies each from four pigs based on different sample sizes. Diversity is calculated according to both Shannon and Simpson’s diversity indices. Black dots show the mean estimates, and bars show the 95% confidence intervals obtained from 10,000 bootstrap iterations. (PPTX 5362 kb)
Additional file 4: Dendrograms showing the relatedness of *E. coli* strains within each pen. (PPTX 5421 kb)
Additional file 5: Dendrograms showing the relatedness of *E. coli* strains within each group of strains (isolated from media with or without antibiotic). (PPTX 5392 kb)

## Abbreviations

*H′*: Shannon diversity index
N: Number
N col: Number of colonies
PFGE: Pulsed field gel electrophoresis
RAPD: Randomly amplified polymorphic DNA
REP-PCR: Repetitive extragenic palindromic-polymerase chain reaction
