# Supplementary figures and images for: Genotype variation and genetic relationship among Escherichia coli from nursery pigs located in different pens in the same farm
Source: BMC Microbiol. 2017 Jan 5;17:5. doi: 10.1186/s12866-016-0912-3 (PMC5217417; doi:10.1186/s12866-016-0912-3)

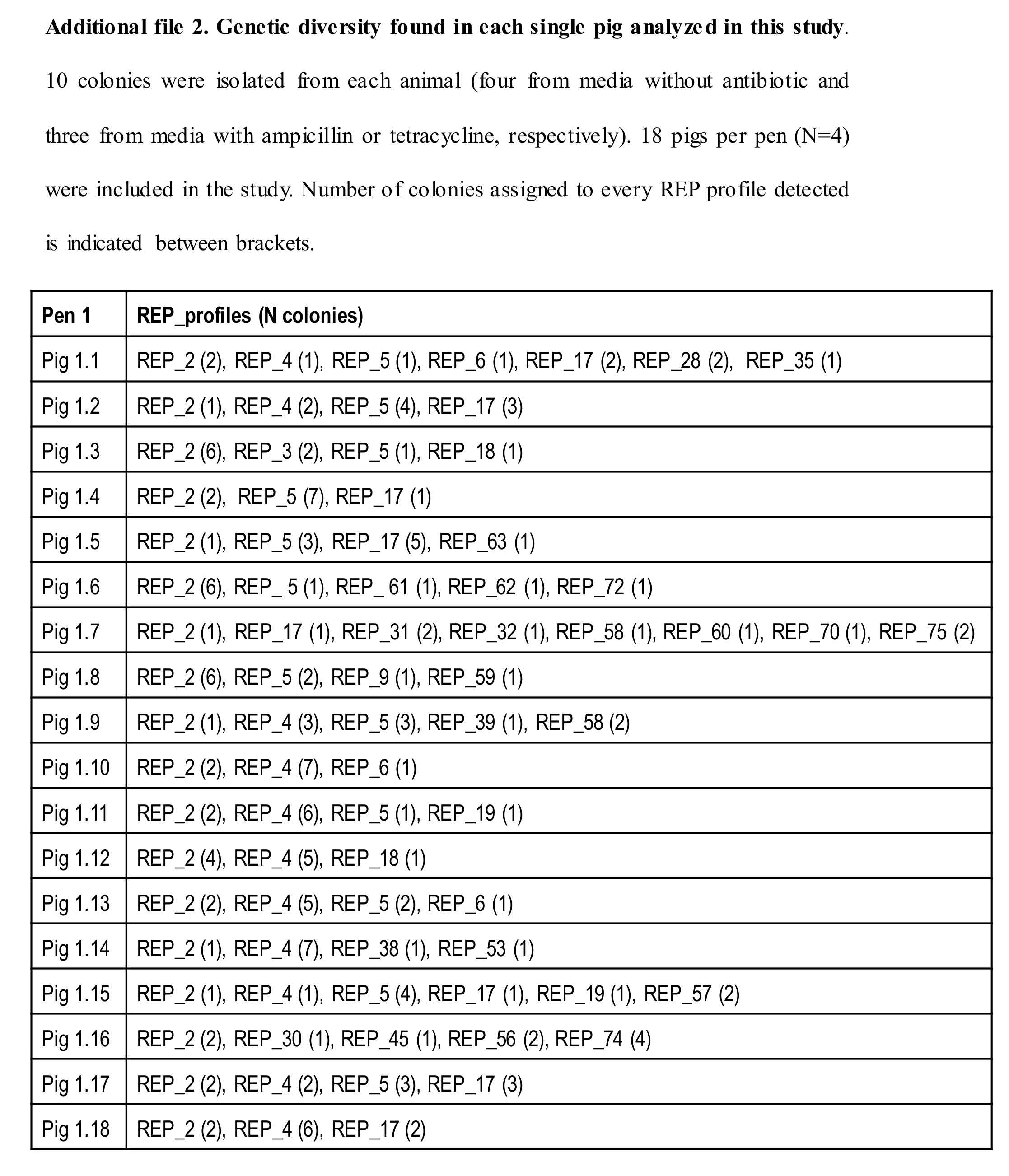

Supplement: Additional file 2: — Genetic diversity found in each single pig analyzed in this study. 10 colonies were isolated from each animal (four from media without antibiotic and three from media with ampicillin or tetracycline, respectively). 18 pigs per pen (N = 4) were included in the study. Number of colonies assigned to every REP profile detected is indicated between brackets. (TIFF 662 kb) [file 12866_2016_912_MOESM2_ESM.tiff]
