# Supplementary material for: Genotype variation and genetic relationship among Escherichia coli from nursery pigs located in different pens in the same farm
Source: BMC Microbiol. 2017 Jan 5;17:5. doi: 10.1186/s12866-016-0912-3 (PMC5217417; doi:10.1186/s12866-016-0912-3)
Supplement: Additional file 3: — The distribution of E. coli diversity obtained using a non-parametric bootstrap procedure to calculate diversity of a randomly chosen subset of 50 colonies each from four pigs based on different sample sizes. Diversity is calculated according to both Shannon and Simpson’s diversity indices. Black dots show the mean estimates, and bars show the 95% confidence intervals obtained from 10,000 bootstrap iterations. (PPTX 5362 kb) [file 12866_2016_912_MOESM3_ESM.pptx]

## Slide 1
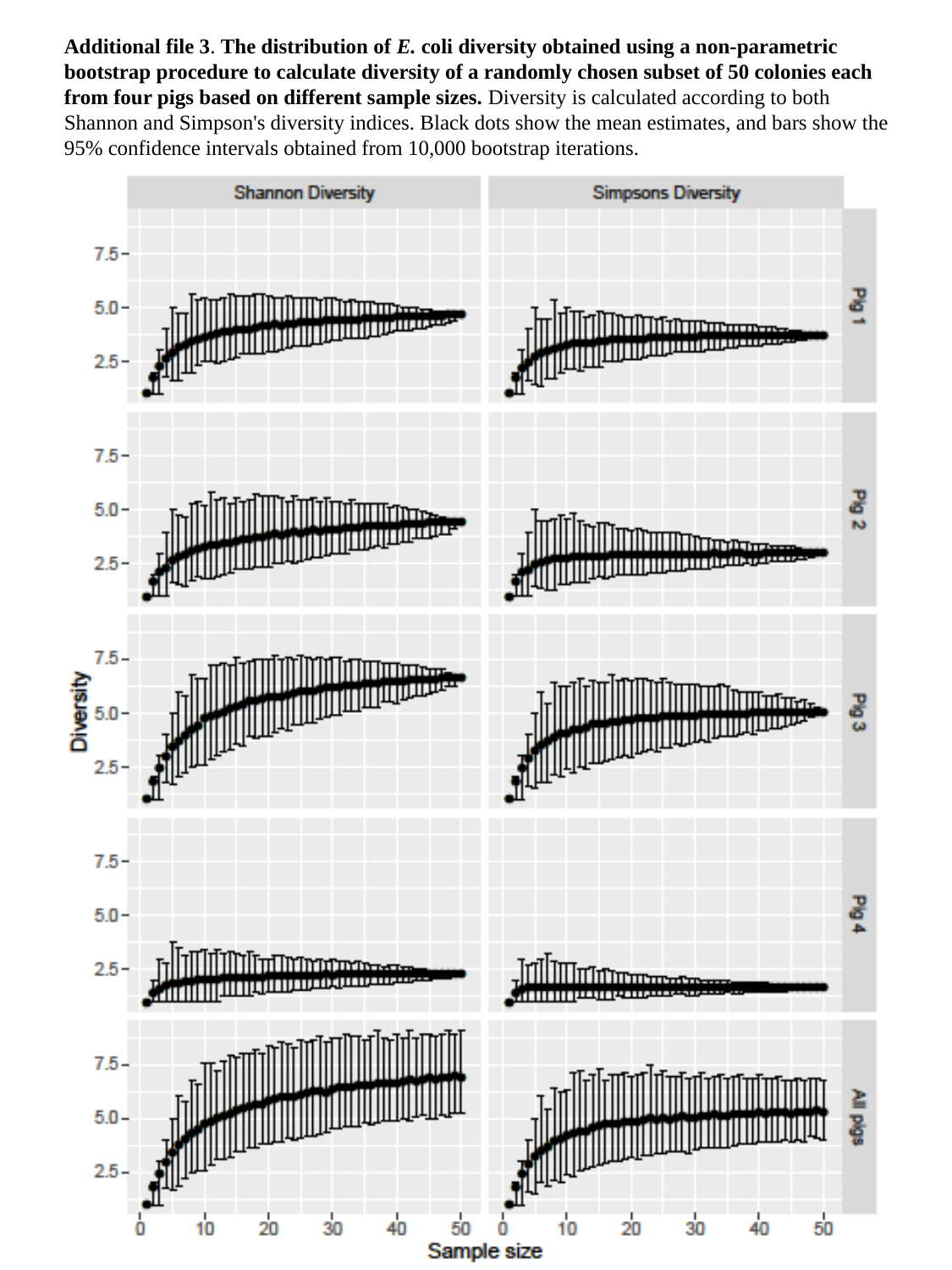

Additional file 3. The distribution of E. coli diversity obtained using a non-parametric bootstrap procedure to calculate diversity of a randomly chosen subset of 50 colonies each from four pigs based on different sample sizes. Diversity is calculated according to both Shannon and Simpson's diversity indices. Black dots show the mean estimates, and bars show the 95% confidence intervals obtained from 10,000 bootstrap iterations.
