# Supplementary material for: Genotype variation and genetic relationship among Escherichia coli from nursery pigs located in different pens in the same farm
Source: BMC Microbiol. 2017 Jan 5;17:5. doi: 10.1186/s12866-016-0912-3 (PMC5217417; doi:10.1186/s12866-016-0912-3)

## Slide 1
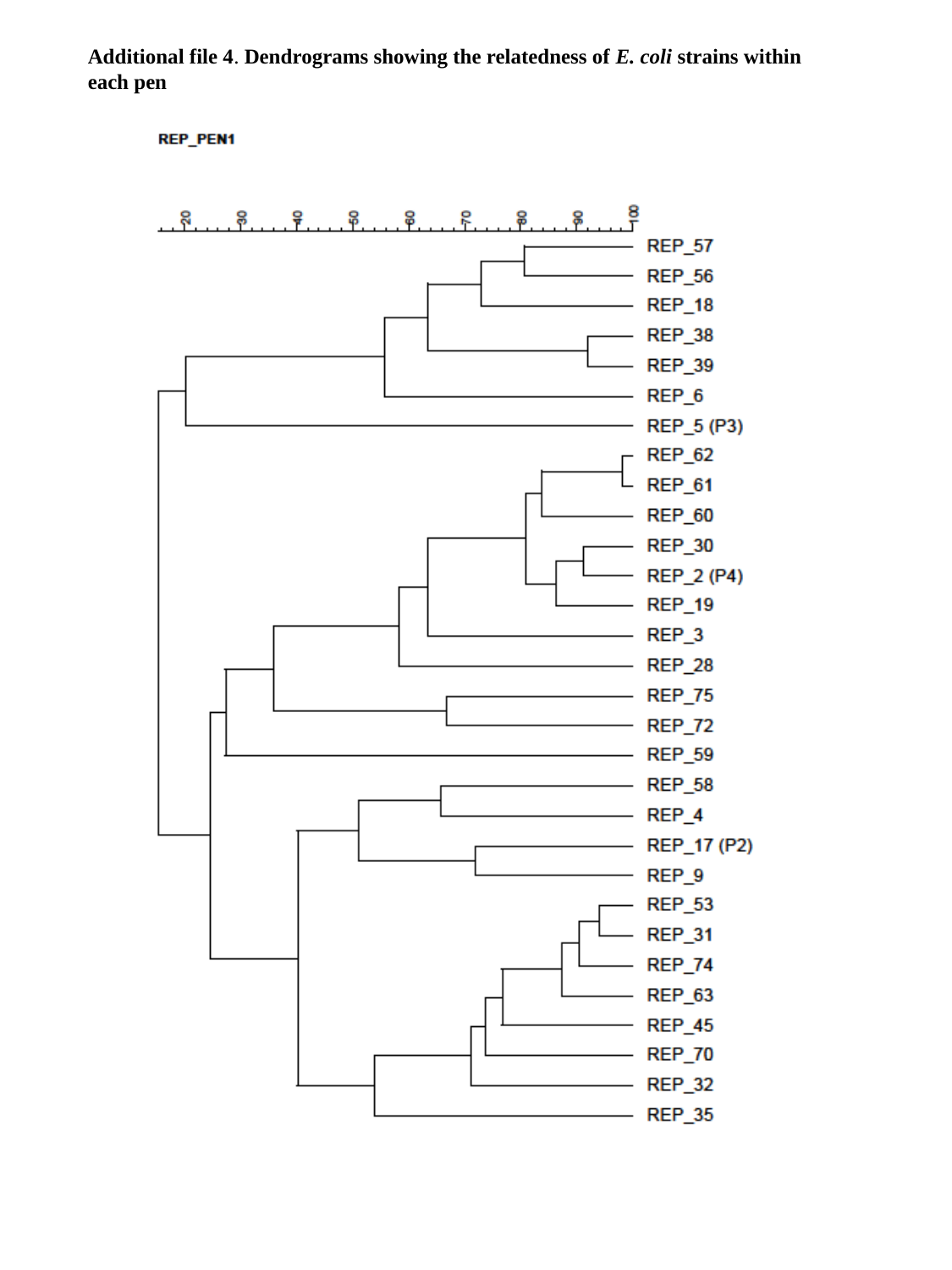

Additional file 4. Dendrograms showing the relatedness of E. coli strains within each pen

## Slide 2
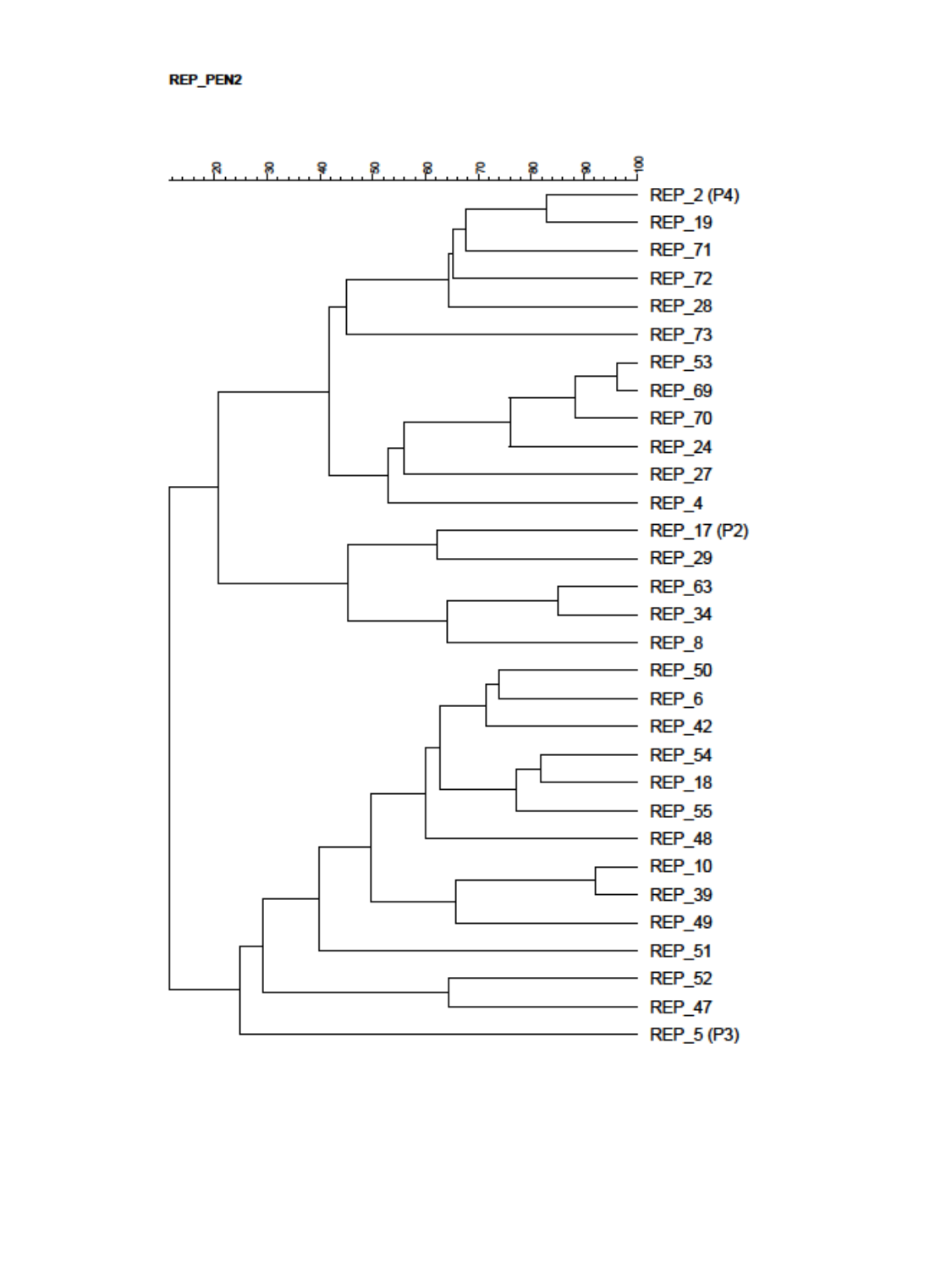

## Slide 3
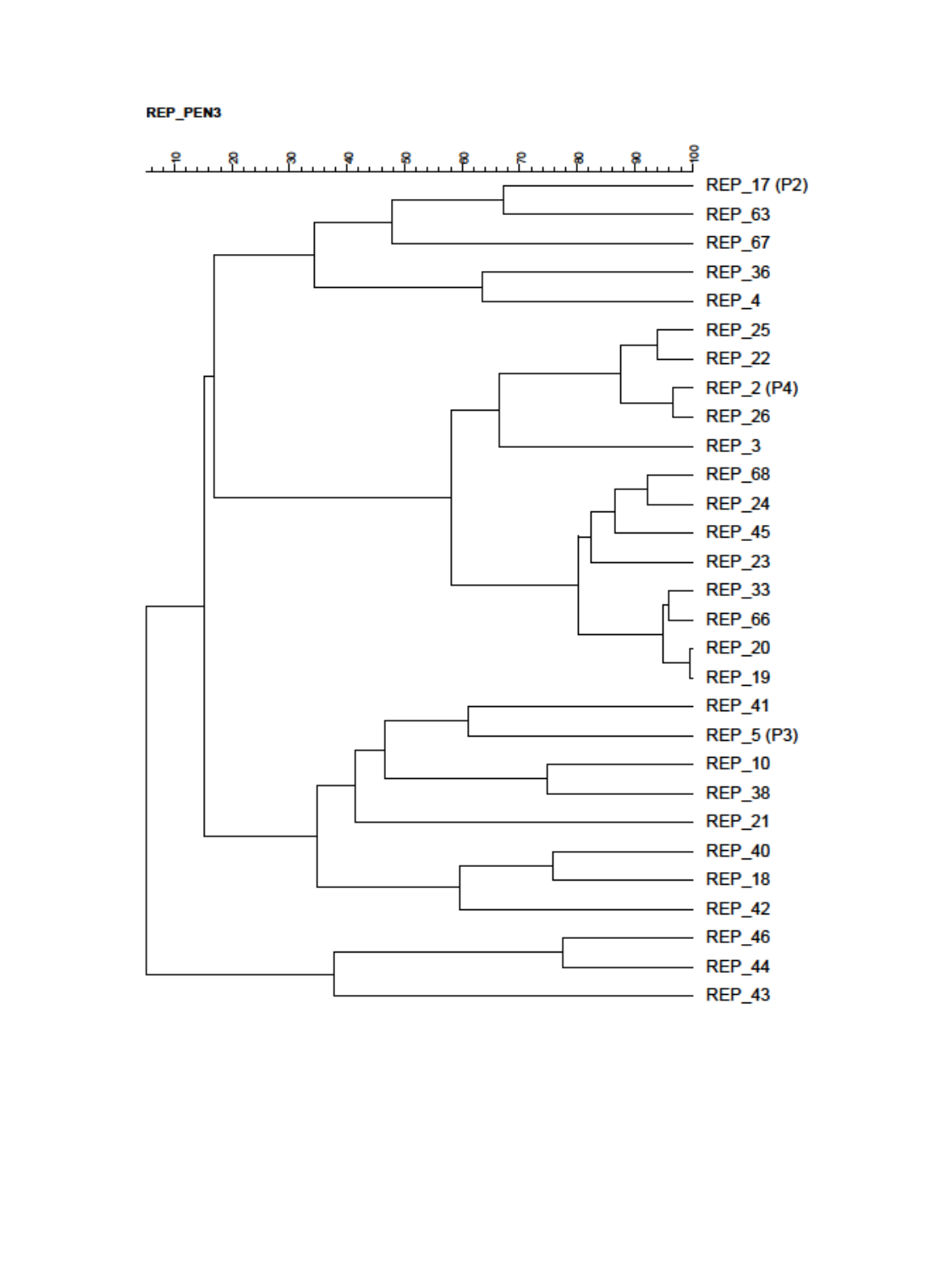

## Slide 4
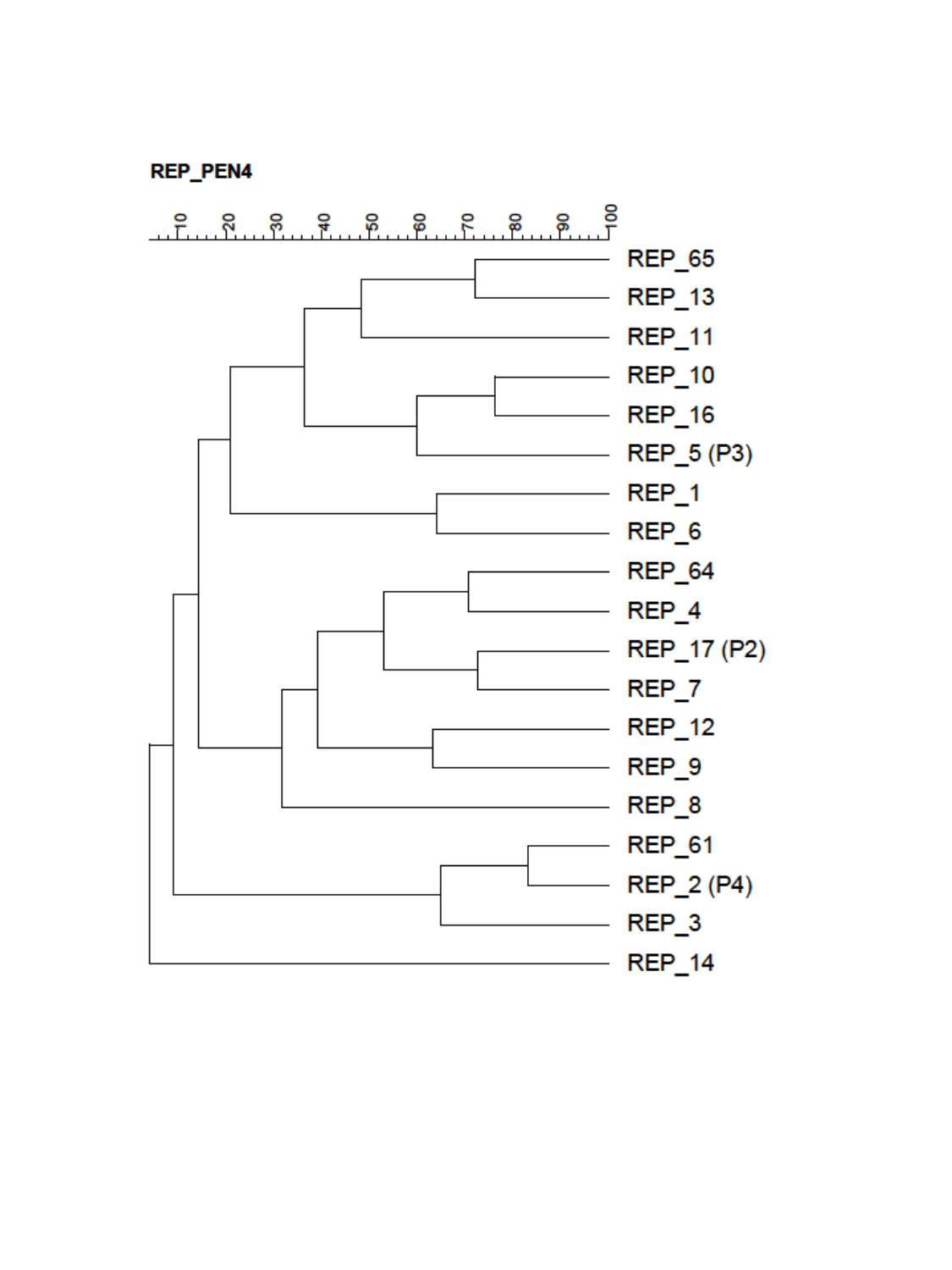

Supplement: Additional file 4: — Dendrograms showing the relatedness of E. coli strains within each pen. (PPTX 5421 kb) [file 12866_2016_912_MOESM4_ESM.pptx]
