# Supplementary material for: Genotype variation and genetic relationship among Escherichia coli from nursery pigs located in different pens in the same farm
Source: BMC Microbiol. 2017 Jan 5;17:5. doi: 10.1186/s12866-016-0912-3 (PMC5217417; doi:10.1186/s12866-016-0912-3)

## Slide 1
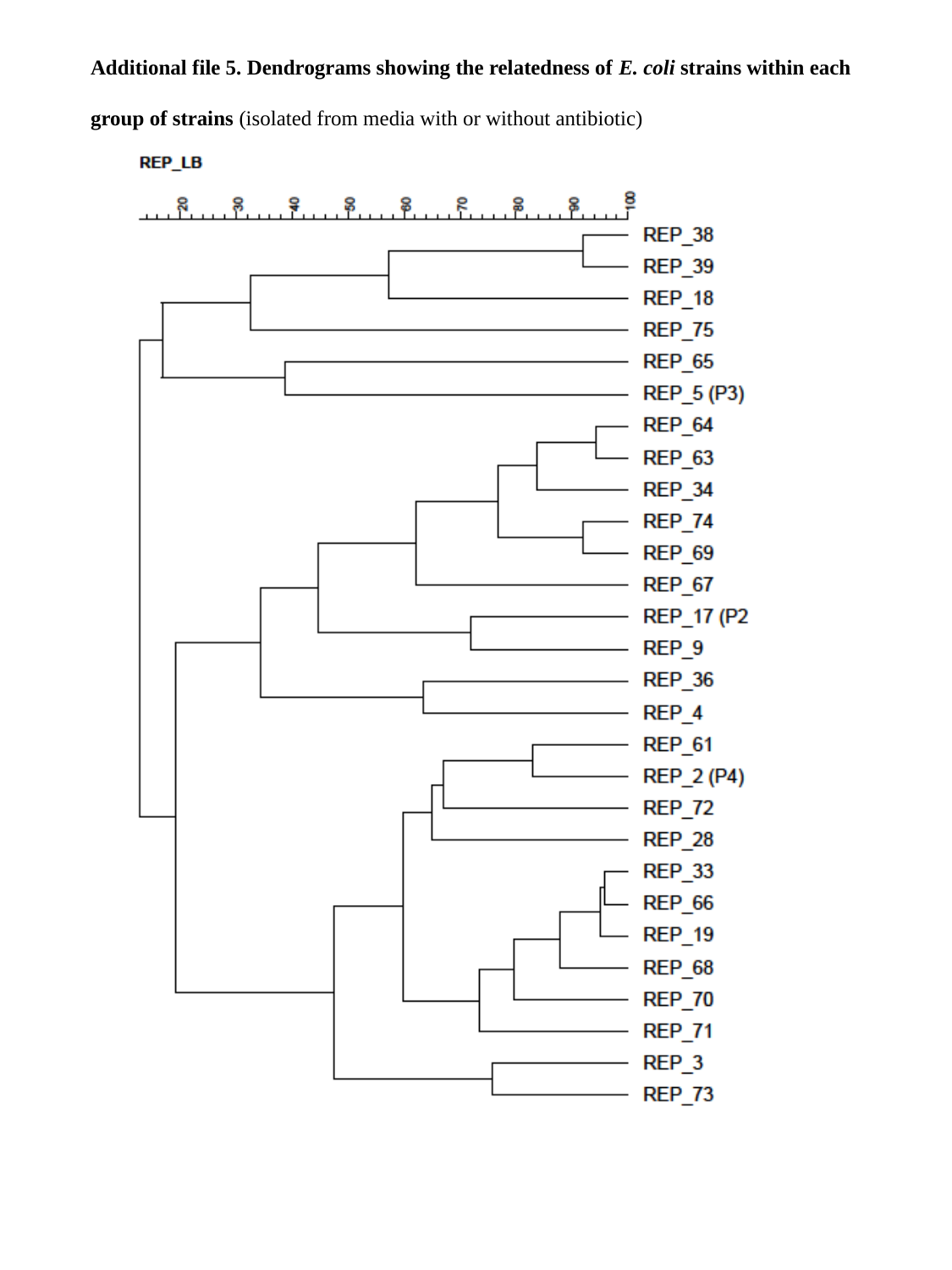

Additional file 5. Dendrograms showing the relatedness of E. coli strains within each group of strains (isolated from media with or without antibiotic)

## Slide 2
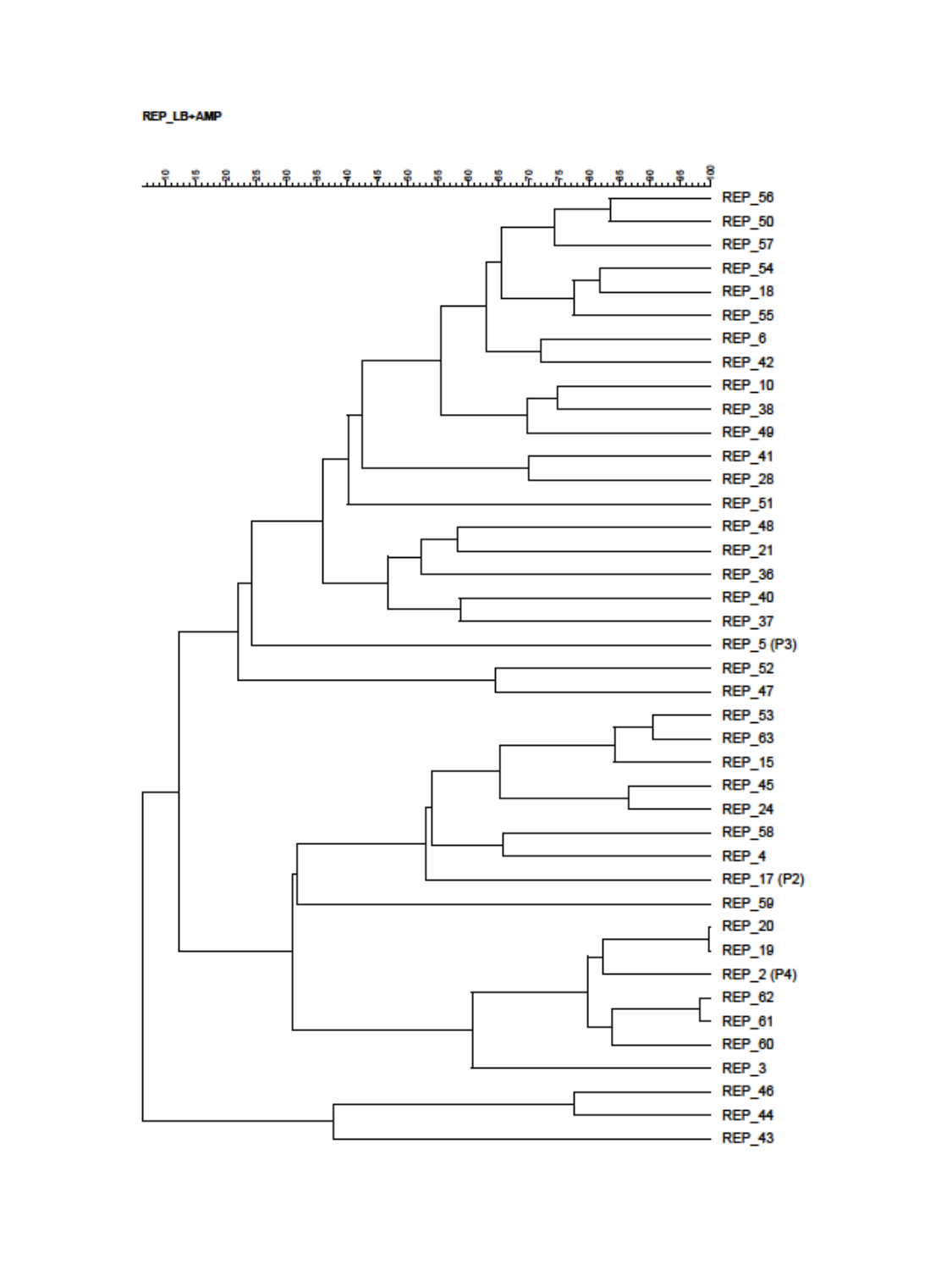

## Slide 3
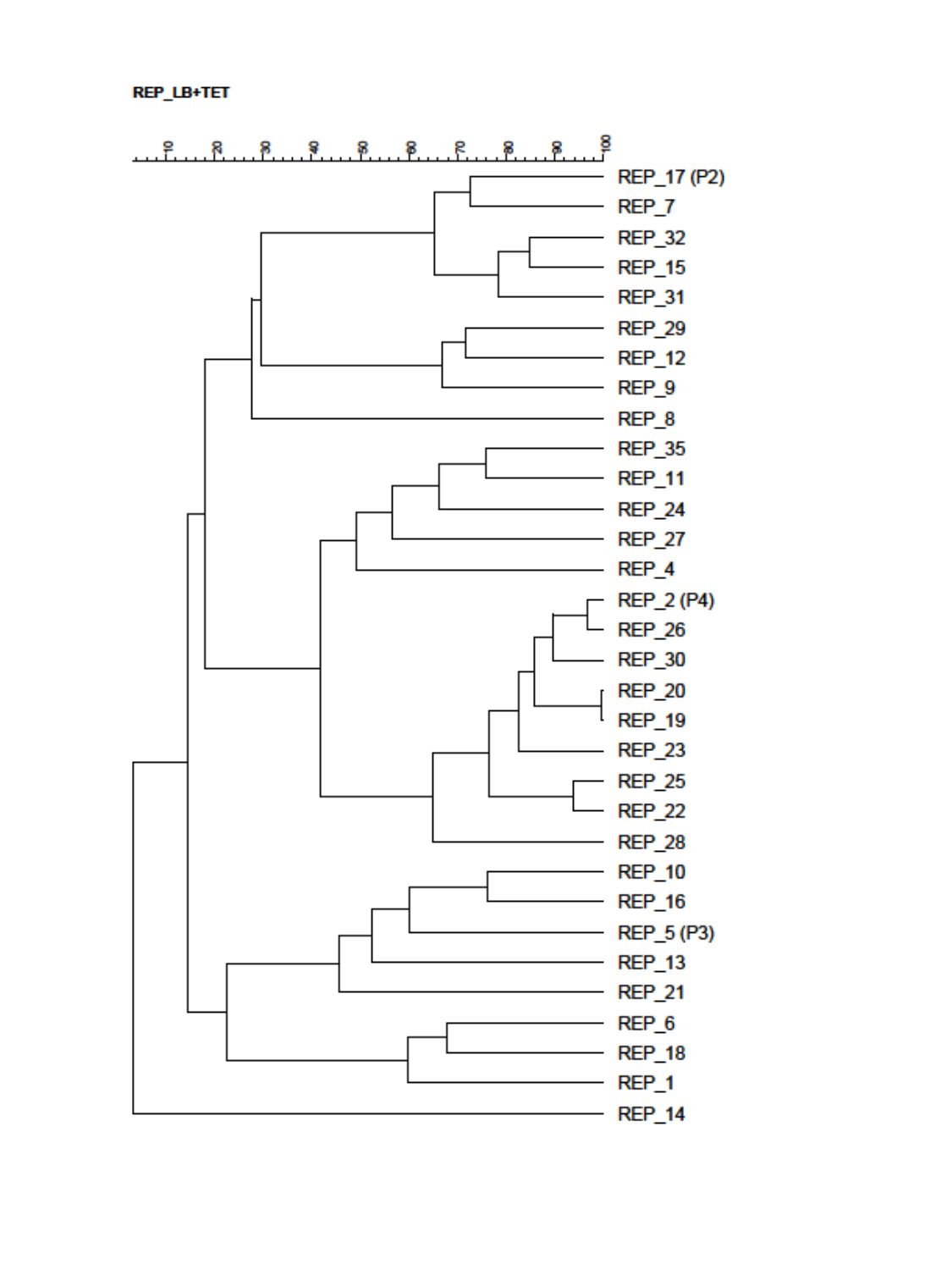

Supplement: Additional file 5: — Dendrograms showing the relatedness of E. coli strains within each group of strains (isolated from media with or without antibiotic). (PPTX 5392 kb) [file 12866_2016_912_MOESM5_ESM.pptx]
